# Nexus between postpartum depression and exclusive breastfeeding practices among lactating mothers in Assosa Town, West Ethiopia

**DOI:** 10.3389/fnut.2024.1357264

**Published:** 2024-04-23

**Authors:** Abdulfeta Abdurehim, Yabsra Melaku, Habtamu Hassen, Hassen Mosa, Musa Jemal, Mohammed Jemal Abawari, Abdurezak Kemal, Tofik Mohammed, Bayise Biru, Emana Alemu, Anwar Seid Ali, Bekri Mohammed, Behre Dari Mosa, Shemsu Kedir, Kalkidan Hassen Abate

**Affiliations:** ^1^Benshangul-Gumz Regional Health Bureau, Assosa, Ethiopia; ^2^Department of Nutrition and Dietetics, Institute of Health, Jimma University, Jimma, Ethiopia; ^3^Department of Emergency Medical Care, Hossana College of Health Sciences, Hossana, Ethiopia; ^4^Department of Midwifery, College of Medicine and Health Sciences, Werabe University, Werabe, Ethiopia; ^5^Department of Public Health, College of Medicine and Health Sciences, Werabe University, Werabe, Ethiopia; ^6^Department of Health Behaviour and Society, Institute of Health, Jimma University, Jimma, Ethiopia; ^7^Department of Public Health, College of Medicine and Health Sciences, Wolkite University, Wolkite, Ethiopia; ^8^Department of Internal Medicine, College of Medicine and Health Sciences, Arbaminch University, Arbaminch, Ethiopia; ^9^Department of Public Health, Institute of Health Sciences, Wollega University, Nekemte, Ethiopia; ^10^Ethiopian Public Health Institute, Addis Ababa, Ethiopia; ^11^Department of Nursing, College of Medical Health Science, Samara University, Samara, Ethiopia; ^12^Department of Public Health, College of Medicine and Health Sciences, University of Gonder, Gonder, Ethiopia; ^13^Laboratory Directorate, Worabe Comprehensive Specialized Hospital, Werabe, Ethiopia

**Keywords:** exclusive breastfeeding, PPD, predictors, Assosa Town, nexus

## Abstract

**Background:**

Across the globe, breastfeeding stands out as a highly effective strategy for reducing infant and child morbidity and mortality. Concurrently, postpartum depression (PPD) emerges as a notable public health issue, adversely affecting both exclusive breastfeeding (EBF) practices for infants and the fulfillment of parenting roles. Despite the lack of substantial evidence in Ethiopia and the specific study areas, indicating the association between PPD and EBF practices, this study endeavors to fill this gap. The primary objective is to examine the correlation between PPD and EBF practices, along with exploring other pertinent factors, in Assosa Town, West Ethiopia.

**Methods:**

A community-based cross-sectional study was carried out from 7 March to 5 April 2019. The study involved the recruitment of 462 participants through a systematic random sampling method. Data collection was facilitated by conducting a structured and pre-tested questionnaire. To screen for PPD, we used the Edinburgh Postnatal Depression Scale (EPDS) tool. This tool, EPDS, was used solely as a screening tool and not for diagnostic purposes. The collected data were entered into Epi-Data version 3.1 and subsequently exported to SPSS version 24 for comprehensive statistical analysis. Bivariate and multivariate logistic regression analyses were performed to assess the association between independent variables and dependent variables. Odds ratios, along with their 95% confidence intervals (CIs), were calculated to ascertain the presence and strength of any associations. Statistical significance was acknowledged at a *p*-value of <0.05.

**Results:**

The overall prevalence of EBF practices was found to be 58.2% (95% CI: 51.4–65.7), while the prevalence of PPD was 18.7% (95% CI: 15.94–26.7). Among mothers without PPD, the prevalence of EBF practices was notably higher at 62.4% (95% CI: 55.9–65.2%) compared to mothers experiencing PPD, where the prevalence was 31.3% (95% CI: 24.7–32.5%). Mothers who experienced PPD exhibited 51% reduced odds of practicing EBF compared to their counterparts (AOR = 0.49. 95% CI: 0.25–0.8). Furthermore, factors such as having a higher family monthly income (AOR = 8.7, 95% CI: 4.2–17.2), being multiparous (AOR = 5.8, 95% CI 4.9–10.8), attending antenatal care (ANC) visits (AOR = 4.9, 95% CI: 3.4–14.1), opting for vaginal delivery (AOR = 9.8, 95% CI: 5.6–17.4), and receiving husband’s support (AOR = 5.3, 95% CI: 4.6–12.7) demonstrated a statistically significant positive association with EBF practices.

**Conclusion:**

In this study, a substantial number of mothers demonstrated suboptimal EBF practices during the first 6 months of their infants’ lives. Consequently, the findings underscore a clear association between PPD and EBF. Thus, it is imperative to intensify efforts in the early detection and treatment of PPD, enhance household income, advocate for ANC, and encourage active husband involvement to bolster EBF practices.

## Introduction

The failure to adhere to exclusive breastfeeding (EBF) has resulted in over 1.4 million deaths globally within the first 6 months of life, with 41% occurring in sub-Saharan Africa and 34% in South Asia (1–3). As reported by the Ethiopian Demographic Health Survey (EDHS) in 2016, the overall prevalence of EBF practices was 58%. Additionally, the median duration of EBF in the Benishangul-Gumuz region was determined to be 4.6 months. The 2016 EDHS findings also revealed neonatal, infant, and under-5 mortality rates of 29, 48, and 67 deaths per 1,000 live births, respectively (4). Despite variations in EBF practice prevalence across different regions, the aggregated prevalence of EBF in Ethiopia was calculated at 59.3% (5).

Different studies showed that there is inconclusive and varying EBF coverage and contributing factors (6–8). Recent studies have suggested an association between symptoms of postpartum depression (PPD) and the early discontinuation of EBF (9). PPD is a non-psychotic form of depression that manifests within the first 12 months after childbirth. The symptoms closely resemble those of depression, which can affect anyone, regardless of childbirth (10). It is a form of depression that impacts approximately 1 in 10 new mothers within the initial year following childbirth. It has the potential to adversely affect a new mother’s health and her capacity to provide nurturing care to her infant.

The burden of PPD in African American women ranges from 10 to 15% (11). While research on the perinatal mental health of women in low and middle-income countries has gained attention more recently, it has historically taken a backseat to the primary focus on preventing pregnancy-related complications (12). The pooled prevalence of PPD in 53 low- and middle-income countries was 19% (13). Moreover, 18.3% were reported by the two African systematic reviews (14), which is as high as those reported in other developed countries (15). Recent Ethiopian studies have reported PPD prevalence to range from 12.2 to 22.1% in rural areas (16, 17) and 9.27 to 33.2% (18–21) in urban areas.

PPD is the most common complication of childbirth (22), a leading cause of disability (23), linked to a weakened mother-infant bond, potentially leading to prolonged effects on the emotional and cognitive growth of the infant (24) worldwide. It contributes to increased costs for the healthcare system and can have a negative impact on the workforce economy. Moreover, depressed women have been reported to face challenges in efficient breastfeeding, exhibit reduced utilization of available health services (25, 26), experience negative postnatal birth experiences (27), and demonstrate negative impacts on maternal interaction with their newborns (28). In light of these multifaceted challenges, addressing PPD and fostering EBF not only bolsters maternal and infant wellbeing but also has economic implications for the healthcare sector and the broader economy (29). Noteworthy initiatives underscore the commitment to maternal and child health in Ethiopia. The establishment of the National Nutrition Programme and the National Guideline on Adolescent, Maternal, Infant, and Young Child Nutrition in 2013 (30) symbolizes a concerted effort to uphold international standards, foster appropriate feeding practices and caregiving approaches, and foster a brighter future for mothers and children in the country.

Enhancing nutritional education, integrating maternal mental health into regular maternal healthcare services, providing psychotherapy support while breastfeeding may have more impact than routine counseling, offering financial assistance to mothers with low income, and providing health education to multiparous women are crucial interventions to enhance appropriate infant feeding practices (31, 32).

While Ethiopia has witnessed a gradual increase in EBF prevalence, suboptimal EBF practices persist as a significant contributor to child mortality in the country. Concurrently, PPD emerges as a notable public health concern, negatively impacting a new mother’s health and her ability to provide nurturing care to her infant. Despite the lack of substantial evidence in Ethiopia and the study areas regarding the impact of PPD on EBF practices, this study endeavors to fill this gap. EBF has been encouraged for mothers who deliver at healthcare facilities. Yet, diagnosing PPD in the community remains challenging without severe cases being identified by healthcare professionals at these facilities. This study was conducted to shed light on this community issue. The primary objective is to investigate the interconnected relationship between PPD and EBF practices, along with exploring other pertinent factors, in Assosa Town, Benishangul-Gumuz Region, West Ethiopia.

## Methods and materials

This community-based cross-sectional study was conducted in Assosa Town, Benishangul-Gumuz Region, West Ethiopia, from 7 March to 5 April 2019. Assosa zone is one of the three zones in Benishangul-Gumuz Regional State. It is situated 665 kilometers from Addis Ababa, the capital city of Ethiopia, with an elevation ranging from 580 to 1,668 meters above sea level. The Mao-Komo special woreda borders Assosa Town to the south, Sudan to the west, and the Kamashi zone to the northeast. The zone’s population, consisting of various ethnic groups, was estimated to be 477,852 people based on the 2007 census data, with 6,000 identified as breastfeeding women. In the latest data available, it was indicated that approximately 151,890 are women of reproductive age. Among them, 804 mothers gave birth at a health facility (24).

### Source/study population, and inclusion/exclusion criteria

The source population encompassed all lactating mothers with children under the age of 1 year residing in Assosa Town.

The inclusion criteria were lactating mothers with children under the age of 1 year residing in Assosa Town for the last 6 months. Whereas, those who were seriously ill or unable to communicate were excluded from the study.

### Sample size determination

A final sample size of 462 was calculated using a single population proportion formula by considering the following assumptions: drawn from a study conducted in Debre Birhan: 70.8% estimated proportion of EBF (33), 5% margin error, 95% confidence interval (CI), 5% allowance for non-response rate, and 1.5 design effect and correction formula with a source population of 6,000 (<10,000) at the study period.

### Sampling procedures

The study participants were chosen through a systematic random sampling method. Initially, three kebeles in Assosa Town were selected using a lottery method. Prior to the actual data collection period, information on mothers who had given birth within the previous 12 months was gathered from family folders and delivery registration books in each Woreda Health Office of the selected kebeles to construct a sampling frame.

Each household was consecutively given a corresponding house number according to the sampling frame and k-value calculated. Since the sampling interval was calculated to be four, every fourth interval was used to enroll the study participants. If a household had more than one lactating mother with a history of birth in the previous 12 months, one of the women was recruited using the lottery method. Probability proportional to size allocation was used to allocate the study participants in each Kebele.

A structured and pre-tested questionnaire was used to collect the data. The questionnaire was developed based on tools that were applied in other related studies (6–8, 34) and the Edinburgh Postnatal Depression Scale (EPDS) (35). The questionnaire’s validity was checked through the application of relevant validity criteria (content validity). Moreover, this tool was validated by the study conducted in Addis Ababa, Ethiopia (36). Furthermore, the EPDS was used solely as a screening tool and not for diagnostic purposes.

After the pre-test, a reliability test was performed, and the questionnaire’s internal reliability (coefficient alpha) was found to be 0.87. For data collection and supervision, six midwives were employed, four with diplomas and two with bachelor’s degrees who were fluent in the local language. We employed midwives for the sake of giving clinical advice to mothers who have mixed feeding and depression as well, due to ethical concerns. The data quality was maintained throughout the study. Initially, the questionnaire was translated into Amharic (the local language) and back-translated into English to ensure consistency before data collection started. Both data collectors and supervisors also received 2 days of training on the study’s goals, data collection methods, and interview protocol. Furthermore, the questionnaire was pre-tested on 5% of the total sample size, which included participants from outside the studied area. The questionnaire’s completeness, accuracy, and applicability were thoroughly examined. In cases where mothers were not present during the initial visit by data collectors, revisits were conducted two or three times, with a gap of 2–3 days between visits if possible. The investigators and supervisor consistently reviewed completed questionnaires to ensure the completeness and accuracy of the details. Additionally, all data underwent meticulous entry and cleaning procedures before the analysis.

### Study variables

The principal outcome variable in this study was EBF practice. The primary independent variable was PPD, while secondary independent variables encompassed socio-demographic factors, including the age of mothers, age of children, marital status, wealth status, husband support, and educational status. Additionally, obstetric history variables, such as gravida, antenatal care (ANC) visit, place of delivery, mode of delivery, and postnatal care, were considered.

### Measurements

#### EBF

In this study, self-reporting was used to determine EBF. “EBF” was considered if the mother responded “yes” to the question is your baby solely receives breast milk, no other liquids or solids are offered, including water (except for oral rehydration solution, vitamin, mineral, or medicine drops, and syrups) during the first 6 months of the infant’s life (35).

Depression that begins within 1 month of childbirth and lasts more than 2 weeks is known as PPD. During the EBF period, a self-reported diagnosis of depression was used to assess the depressive status of the mother. It comprised 21 items, each measured on a scale of 0 to 3, and the total sum was 0 to 63. Women with an EPDS ≥10 were considered to have PPD (35, 37).

### Statistical analysis

The SPSS version 24 software was used for data entry and computing statistical analysis. Descriptive statistics such as socio-demographic characteristics, obstetric history, breastfeeding, and PPD are presented in a table, figure, and standard deviation to summarize the findings. Initially, bivariable binary logistic regression analysis was used to detect candidate variables for multivariable binary logistic regression analysis. Then, all independent variables having a *p-*value of ≤0.25 in the bivariate logistic regression analysis were entered into the multivariable logistic regression analysis to find out the independent factors. An odds ratio with a 95% CI was used to decide the strength and direction of the association between the dependent and independent variables. To ensure the necessary assumptions for multivariable binary logistic regressions, a Hosmer–Lemeshow goodness-of-fit test was used and met. Finally, the significance level was declared at a *p*-value of *<*0.05.

### Ethics approval and consent to participation

The study protocol was approved by the Institutional Review Board of Jimma University, the Institute of Health, and the Faculty of Health Science. Additionally, all relevant authorities in the Benishangul-Gumuz Regional Health Bureau gave their authorization. Before enrollment in the study, participants provided written informed consent. A right thumbprint was used as a signature for participants who were unable to write. For participants under the age of 18, parental or legal guardian consent was obtained. The study was fully voluntary, and participants were told of their right to withdraw at any time. Data were collected confidentially, and it was de-identified, de-linked, and stored in a safe location.

## Results

### Sociodemographic and obstetric characteristics

The mean age of the respondents was 28.4 ± 4.7 years with a response rate of 97.4%. The majority of respondents were married 429 (95.3%), 146 (32.4%) were Amhara in ethnics, 243 (60.3%) were housewives, and 180 (40%) had no formal education. In terms of obstetric characteristics, 335 (74.4%) of the respondents were primiparous, while 197 (43.8%) had ANC visits. The majority of the respondents, 431 (95.8%), gave birth vaginally. Other socio-demographic and obstetric characteristics are presented in Table 1.

**Table 1:** Socio-demographic and obstetric characteristics of the participants in Assosa Town, West Ethiopia, 2019.

Variables	Frequency (*n* = 450)	Percent
*Age of the mothers in years*		
≤25	147	32.7
26–30	166	36.9
≥31	137	30.4
*Ethnicity*		
Amhara	146	32.4
Oromo	117	26.0
Berta	145	32.2
Others	42	9.4
*Marital status*		
Married	429	95.3
Others	21	4.7
*Age of the child in months*		
0–3	165	36.7
4–6	135	30.0
7–12	150	33.3
*Maternal educational status*		
No formal education	180	40
Primary education	145	32.3
Secondary education	85	18.8
College and above	40	8.9
*Mothers’ occupation*		
Government employee	116	25.8
Merchant	103	22.9
Housewife	200	44.5
Daily laborer	31	6.8
*ANC visits*		
Yes	197	43.8
No	253	56.2
*Postnatal care follow-up*		
Yes	277	61.6
No	173	38.4
*Place of delivery*		
Health facility	331	73.6
Home	119	26.4
*Mode of delivery*		
Vaginal delivery	431	95.8
Cesarean section	19	4.2
*Gravidity*		
Primiparous	335	74.4
Multiparous	115	25.6
*Having the husband’s support*		
Yes	203	45.1
No	247	54.9

### Breastfeeding practices

The prevalence of EBF during the first 6 months of life was observed to be 58.2% (95% CI: 51.4–65.7). The majority of respondents, 386 (85.8%), replied they were informed about EBF at some point, 304 (67.6%) of them started breastfeeding within 1 h of birth, and half, 225 (50%), said they got information from health care providers. Of all respondents, approximately half (217 (48.2%)) expressed and discarded their colostrum. In terms of EBF frequency, the majority, 325 (72.2%), of the respondents gave breast milk to their infant eight times and above per 24 h (Table 2).

**Table 2:** Breastfeeding practice-related characteristics of the participant in Assosa Town, West Ethiopia, 2019.

Variables	Frequency (*n* = 450)	Percent
*Practiced EBF*		
Yes	262	58.2
No	188	41.8
*Ever heard about EBF*		
Yes	386	85.8
No	64	14.2
*Expressed and discarded colostrum*		
Yes	217	48.2
No	233	51.8
*Started first breastfeeding within one hour*		
Yes	304	67.6
No	146	32.4
*Know the exact duration of EBF*		
Yes	286	63.6
No	164	36.4
*Know the importance of EBF*		
Yes	448	99.6
No	2	0.4
*Frequency of breastfeeding*		
< 8	125	27.8
≥ 8	325	72.2
*Source of information about breastfeeding*		
From healthcare providers	225	50.0
Husband	147	32.6
Health extension workers	58	12.9
Others	20	4.5

### Distribution between the level of EBF practices and PPD

**Figure 1:**
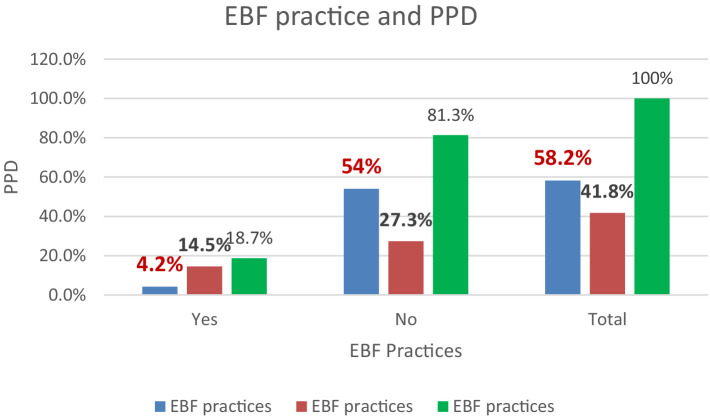
Distribution between the level of EBF practices and PPD.

The prevalence of EBF practices was higher among mothers without PPD (54%), than among postpartum depressed mothers (4.2%) (Figure 1).

### The scale of PPD score

**Figure 2:**
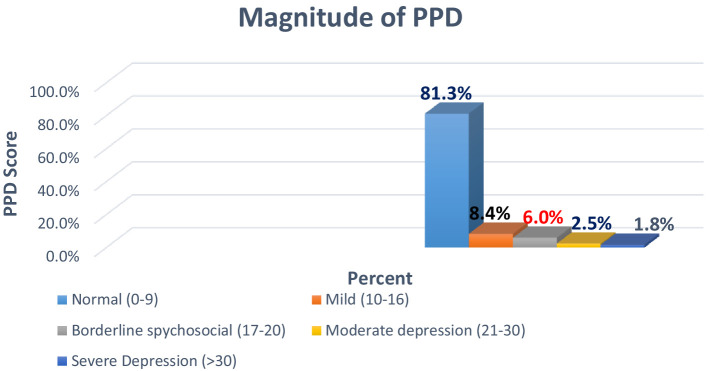
Magnitude of PPD among lactating mothers in Assosa Town, West Ethiopia, 2019.

The overall prevalence of PPD in the study area was 18.7% [95% CI: 15.94–26.6] (Figure 2) based on the EPDS screening tool.

### PPD and other factors associated with EBF practices

In this study, mothers who experienced PPD had 86% reduced odds of EBF their babies than their counterparts (AOR = 0.14. 95% CI: 0.12–0.8) The outcomes of multivariate logistic regression showed that having a medium level of income, being multiparous, ANC visits, giving birth by a vaginal route, having a husband’s support, and experiencing PPD were significantly associated factors of EBF.

Mothers from middle-income households had 8.7 times higher odds of practicing EBF in comparison to mothers of lower-income households [AOR = 8.7, 95 CI (4.2–17.2)]. Similarly, the odds of practicing EBF were 9.8 times higher among mothers who gave birth vaginally than those who had a cesarean section [AOR = 9.8, 95% CI (5.6–17.4)]. In addition, mothers who were supported by their husbands were 5.3-folds more likely to practice EBF than mothers who were not supported by their husbands [AOR = 5.3, 95% CI (4.6–12.7)]. Likewise, mothers receiving ANC visits had 4.9 times greater probabilities of practicing EBF in comparison to their counterparts [AOR = 4.9, 95% CI (3.4–14.1)]. Furthermore, the odds of practicing EBF for multiparous mothers were six times higher when compared to primiparous mothers [AOR = 5.8, 95%CI (4.9–10.8)] (Table 3).

**Table 3:** Binary and multivariate logistic regression results for factors associated with exclusive breastfeeding in Assosa Town, West Ethiopia, 2019.

Variables	EBF		(COR 95% CI)	(AOR 95% CI)
	Yes	No		
*Age in years*				
≤25	88	59	0.9 (0.57–3.4)	0.7 (0.4–2.2)
26–30	103	63	1.3 (0.86–9.2)^*^	1.2 (0.73–6.7)
≥31	71	66	Reference	Reference
*Wealth status of the mother*				
Low	121	20	2 (0.8–5)	1.7 (0.6–4.2)
Medium	101	57	9.3 (6.2–27.1)^*^	8.7 (4.2–17.2)^**^
High	40	111	Reference	Reference
*Gravidity*				
Multiparous	231	104	6 (3.7–9.6)^*^	5.8 (4.9–10.8)^**^
Primiparous	31	84	Reference	Reference
*ANC visits*				
Yes	197	64	5.9 (3.8–18.7)^*^	4.9 (3.4–14.1)^**^
No	65	124	Reference	Reference
*Having the husband’s support*				
Yes	203	70	5.6 (5.3–12.8)^*^	5.3 (4.6–12.7)^**^
No	60	117	Reference	Reference
*Mode of delivery*				
Spontaneous vaginal	243	102	10.7 (6.2–18.6)^*^	9.8 (5.6–17.4)^**^
Cesarean section	19	86	Reference	Reference
*Experiencing postpartum depression*				
Yes	19	65	0.15 (0.11–0.7)^*^	0.14 (0.12–0.8)^**^
No	243	123	Reference	Reference

^*^ = *p* ≤ 0.25, ^**^ = *p* < 0.05.

## Discussion

This study investigated PPD and associated factors related to EBF practices among lactating mothers in Assosa Town. The findings revealed that only 58.2% of mothers practiced EBF for the first 6 months of their child’s life. Moreover, the prevalence of EBF practices was higher among mothers without PPD (54%) than among PPD mothers (4.2%). Notably, the prevalence of EBF in this study was higher than reported rates in studies conducted in Tigray, Addis Ababa, Gojjam, and Somaliland, where EBF rates were documented as 26.9, 29.3, 50.1, and 20.47%, respectively (6–8, 38). The variations in EBF rates across different regions could be attributed to socioeconomic and cultural differences in infant feeding practices. Additionally, disparities may arise from the varying levels of emphasis and effort that countries dedicate to promoting EBF. Cultural norms, healthcare infrastructure, and awareness campaigns can significantly influence the prevalence of EBF in different parts of the world. In contrast, this study found a smaller prevalence of EBF compared with studies carried out in Hawassa, Wolaita, Goba, and Dire Dawa, which were 60.9, 64.8, 71.3, and 81.1%, respectively (39–42). The differences observed in our research findings may be attributed to variations in the study area, sample size, sociocultural factors, economic conditions, and the implementation of EBF practices. These diverse factors can contribute to discrepancies in the prevalence and promotion of EBF in different research contexts.

This study revealed that there was a negative association between EBF and PPD. This finding is in line with studies conducted in Brazil (43), Bangladesh (44), China (45), and the Democratic Republic of the Congo (46). The observed association between PPD and lower EBF rates could be attributed to the potential detrimental impact of PPD on a mother’s self-esteem and memory. These effects may contribute to a shorter duration of breastfeeding and negative postnatal birth experiences, ultimately influencing maternal interaction with their newborns. The psychological and emotional challenges associated with PPD may influence a mother’s ability and willingness to engage in sustained breastfeeding practices. Moreover, women experiencing depression are more likely to have insufficient interactions with their newborns, including reduced touching, sensitivity, and skin-to-skin contact. These factors can increase the likelihood of encountering breastfeeding difficulties. The emotional and psychological state of a depressed mother may impact her ability to establish and maintain positive bonding experiences with her infant, potentially influencing the breastfeeding journey.

We need to acknowledge that the association between PPD and EBF is uncertain. Some studies support that it is PPD that decreases the success of breastfeeding; however, others argue that the discontinuation of breastfeeding leads to a higher risk of experiencing PPD (47–49). Further research and personalized care can shed more light on how these factors interplay and how best to support mothers in managing PPD and achieving successful breastfeeding practices.

In this study, infants born to mothers in the middle wealth index were found to be more likely to practice EBF than those born to mothers in the lowest wealth index. This finding is consistent with previous studies undertaken in Bahir Dar (34), Kenya (50), Ghana (51), Nigeria (52), India (53), and Australia (54). The observed association may be due to higher-income mothers having more access to different information that can help them better understand the benefits of EBF. This finding, however, contradicts the earlier research conducted in Addis Ababa (7), East Gojjam (8), and Bangladesh (44). These variations may be the result of socioeconomic and demographic differences between the regions.

Similarly, mothers who have their husband’s support had higher odds of practicing EBF than their counterparts. The finding is similar to the prior studies conducted in East Gojjam, Ethiopia (8), the United Kingdom (55), Nepal (56), and Vietnam (57). The explanation for this may be that the husband’s involvement is important in family and household decision-making, as well as many other aspects of family life, including infant feeding practices. Moreover, the odds of practicing EBF were higher among multiparous than primiparous mothers. This result is similar to that of the studies undertaken in Malawi (58) and Indonesia (59). The explanation for this may be that multiparous mothers are more likely to practice EBF because they have prior breastfeeding experience.

Likewise, in this study, receiving ANC was positively associated with EBF practice. This finding is supported by studies carried out in Bahir Dar (34), Tanzania (60), Nigeria (52), Malawi (58), and India (53). This indicates that the ANC has a substantial influence on EBF. As a result, mothers who follow an ANC visit may have a strong chance to receive nutritional counseling and education about infant feeding, which includes EBF. Besides, this study revealed that mothers who gave birth vaginally were more likely than those who had a cesarean section to exclusively breastfeed their babies. This finding is consistent with a study from Bahir Dar (34), Hawassa (39), Nigeria (52), and Nepal (57). The reason could be that timely hospital discharge and reconnection with the families of vaginally delivered mothers can make postnatal care simpler and increase the chances of practicing EBF. In addition, the mother’s pain caused her to delay giving the baby breast milk and instead use formula or cow milk as a first option.

The strengths of this study comprise the fact that respondents were enrolled using the probability sampling technique to maintain the representativeness of the study, which made it community-based, and various methods were used to maintain the quality of data.

However, there are some limitations to this study. First, this study used a cross-sectional study design, making causal associations difficult to establish. Second, the result was determined by self-report; recall and social desirability bias may have affected the underestimation or overestimation of the correlation between the dependent and the independent variables. Moreover, the generalizability of the findings to other populations or regions may be limited due to cultural, socioeconomic, and healthcare system differences. Future research could benefit from including a more diverse sample or conducting similar studies in different settings.

## Conclusion

In conclusion, the prevalence of EBF was found to be low, contrasting with international recommendations advocating for EBF for children under the age of 6 months. Additionally, the study identified an association between PPD and EBF practices. These findings underscore the need for targeted interventions and support to promote optimal breastfeeding practices, particularly in the context of maternal mental health. Owing to a medium level of income, being multiparous, having an ANC visit, vaginal delivery, receiving husband’s support, and experiencing PPD were independently associated factors of EBF. Hence, it is imperative to intensify efforts toward early detection and treatment of PPD, enhancing household income, promoting ANC, and encouraging active husband involvement to enhance EBF practices. Additionally, future research with robust study designs should be undertaken to comprehensively explore the diverse underlying factors influencing EBF. This will contribute to a more nuanced understanding and inform targeted strategies to improve breastfeeding practices.

## Data availability statement

The raw data supporting the conclusions of this article will be made available by the authors, without undue reservation.

## Ethics statement

The studies involving humans were approved by Jimma University Institutional Review Board. The studies were conducted in accordance with the local legislation and institutional requirements. The participants provided their written informed consent to participate in this study.

## Author contributions

AbA: Conceptualization, Data curation, Formal analysis, Funding acquisition, Investigation, Methodology, Project administration, Resources, Software, Supervision, Validation, Visualization, Writing – original draft, Writing – review & editing. YM: Formal analysis, Methodology, Writing – original draft, Writing – review & editing. HH: Formal analysis, Supervision, Writing – original draft, Writing – review & editing. HM: Writing – original draft, Writing – review & editing. MJ: Writing – original draft, Writing – review & editing. MA: Writing – original draft, Writing – review & editing. AK: Writing – original draft, Writing – review & editing. TM: Writing – original draft, Writing – review & editing. BB: Writing – original draft, Writing – review & editing. EA: Writing – original draft, Writing – review & editing. AnA: Writing – original draft, Writing – review & editing. BekM: Writing – original draft, Writing – review & editing. BehM: Supervision, Software, Writing – review & editing. SK: Conceptualization, Data curation, Formal analysis, Funding acquisition, Investigation, Methodology, Project administration, Resources, Software, Supervision, Validation, Visualization, Writing – original draft, Writing – review & editing. KA: Conceptualization, Formal analysis, Investigation, Methodology, Supervision, Validation, Writing – original draft, Writing – review & editing.
